# Response to Letter to the editors of Hopkins et al.: Effects of surgical and FFP2/N95 face masks on cardiopulmonary exercise capacity: the numbers do not add up

**DOI:** 10.1007/s00392-020-01749-z

**Published:** 2020-10-09

**Authors:** Sven Fikenzer, Ulrich Laufs

**Affiliations:** Universitatsklinikum Leipzig, Leipzig, Germany

Sirs:

Hopkins discuss (1) the linear relationship between power output and oxygen consumption, (2) the linear relationship between cardiac output during exercise and oxygen consumption (3) the reduction in ventilation and (4) the leakage test.

1. The linear relationship between oxygen consumption and power output in a ramp test is constant but the relationship is not "rigid" [1, 2]. The aerobic capacity in healthy active persons is plausible between 10 and 12 ml/min/W [3, 4]. The values in our study were nm: 11.7 ± 0.7; sm: 11.5 ± 0.7 and ffpm: 10.7 ± 1.1 ml/min/W (ANOVA *p* = 0.005) and are within this range.
2. The literature of Yamaguchi et al. [5] cited by Hopkins describes a relationship between cardiac output and *V*O_2max_ with the following regression equation: *V*O_2_ as *Q* = *K* (*V*O_2_ − *V*O_2r_) + *Q*_r_, where *K*, *V*O_2r_ and *Q*_r_ are the slope of the regression line, the resting *V*O_2_ and the resting *Q*, respectively. *K* is ranging from 5.5 to 10.3 [5]. In contrast to our investigation, the described relationship refers to a submaximal load. It remains to be established whether this regression equation is also valid at maximum load and when using FM. When *K* is calculated in our study at maximum load, it is within the range reported in this reference [5]: nm: 6.9 ± 1.8; sm: 7.5 ± 1.6 and ffpm: 8.5 ± 2.5, ANOVA *p* = 0.008.
3. The increased breathing resistance caused by FM led to the following physical reactions under maximal load: VE sm: − 12.0 ± 12.6%, ffpm: − 23.1 ± 13.6%, *p* = 0.001, tidal volume sm − 9.9 ± 11.3% and ffpm: − 14.4 ± 13.0%, *p* = 0.016, inhalation time sm: + 12 ± 15%, ffpm: + 19 ± 16%, *p* < 0.001, compared to nm. Due to the reduced tidal volume and the extended inhalation time, a further increase of the VE was not possible when using fm. Thus, the increased work of breathing can be considered a relevant additional factor of fatigue.
4. As described in methods, the leak tightness was tested both during forced inspiration and expiration. Furthermore, the breath-by-breath measurements were completely monitored. There was no evidence of leakage during the tests.

Sven Fikenzer and Ulrich Laufs
