# Correction to: Response to Letter to the editors of Hopkins et al.: Effects of surgical and FFP2/N95 face masks on cardiopulmonary exercise capacity: the numbers do not add up

**DOI:** 10.1007/s00392-021-01967-z

**Published:** 2021-11-15

**Authors:** Sven Fikenzer, Ulrich Laufs

**Affiliations:** Universitatsklinikum Leipzig, Leipzig, Germany

## Correction to: Clinical Research in Cardiology (2020) 109:1607 10.1007/s00392-020-01749-z

The article “Response to Letter to the editors of Hopkins et al.: Effects of surgical and FFP2/N95 face masks on cardiopulmonary exercise capacity: the numbers do not add up”, written by Sven Fikenzer and Ulrich Laufs, was originally published Online First without Open Access. After publication in volume 109, issue 12, page 1607 the author decided to opt for Open Choice and to make the article an Open Access publication. Therefore, the copyright of the article has been changed to © The Author(s) 2020 and the article is forthwith distributed under the terms of the Creative Commons Attribution 4.0 International License, which permits use, sharing, adaptation, distribution and reproduction in any medium or format, as long as you give appropriate credit to the original author(s) and the source, provide a link to the Creative Commons licence, and indicate if changes were made.

The images or other third party material in this article are included in the article’s Creative Commons licence, unless indicated otherwise in a credit line to the material. If material is not included in the article’s Creative Commons licence and your intended use is not permitted by statutory regulation or exceeds the permitted use, you will need to obtain permission directly from the copyright holder.

To view a copy of this licence, visit http://creativecommons.org/licenses/by/4.0/.

The original article has been corrected.

